# Association of Smartphone Ownership and Internet Use With Markers of Health Literacy and Access: Cross-sectional Survey Study of Perspectives From Project PLACE (Population Level Approaches to Cancer Elimination)

**DOI:** 10.2196/24947

**Published:** 2021-06-09

**Authors:** Sachiko M Oshima, Sarah D Tait, Samantha M Thomas, Oluwadamilola M Fayanju, Kearston Ingraham, Nadine J Barrett, E Shelley Hwang

**Affiliations:** 1 Duke University School of Medicine Durham, NC United States; 2 Department of Biostatistics & Bioinformatics Duke University School of Medicine Durham, NC United States; 3 Duke Cancer Institute Durham, NC United States; 4 Department of Surgery Duke University School of Medicine Durham, NC United States; 5 Department of Family Medicine and Community Health Duke University School of Medicine Durham, NC United States; 6 Duke Clinical Translation Science Institute Duke University School of Medicine Durham, NC United States

**Keywords:** telehealth, technology, health literacy, access to health care, mobile phone

## Abstract

**Background:**

Telehealth is an increasingly important component of health care delivery in response to the COVID-19 pandemic. However, well-documented disparities persist in the use of digital technologies.

**Objective:**

This study aims to describe smartphone and internet use within a diverse sample, to assess the association of smartphone and internet use with markers of health literacy and health access, and to identify the mediating factors in these relationships.

**Methods:**

Surveys were distributed to a targeted sample designed to oversample historically underserved communities from April 2017 to December 2017. Multivariate logistic regression was used to estimate the association of internet and smartphone use with outcomes describing health care access and markers of health literacy for the total cohort and after stratifying by personal history of cancer. Health care access was captured using multiple variables, including the ability to obtain medical care when needed. Markers of health literacy included self-reported confidence in obtaining health information.

**Results:**

Of the 2149 participants, 1319 (61.38%) were women, 655 (30.48%) were non-Hispanic White, and 666 (30.99%) were non-Hispanic Black. The median age was 51 years (IQR 38-65). Most respondents reported using the internet (1921/2149, 89.39%) and owning a smartphone (1800/2149, 83.76%). Compared with the respondents with smartphone or internet access, those without smartphone or internet access were more likely to report that a doctor was their most recent source of health information (344/1800, 19.11% vs 116/349, 33.2% for smartphone and 380/1921, 19.78% vs 80/228, 35.1% for internet, respectively; both *P*<.001). Internet use was associated with having looked for information on health topics from any source (odds ratio [OR] 3.81, 95% CI 2.53-5.75) and confidence in obtaining health information when needed (OR 1.83, 95% CI 1.00-3.34) compared with noninternet users. Smartphone owners had lower odds of being unable to obtain needed medical care (OR 0.62, 95% CI 0.40-0.95) than nonsmartphone owners. Among participants with a prior history of cancer, smartphone ownership was significantly associated with higher odds of confidence in ability to obtain needed health information (OR 5.63, 95% CI 1.05-30.23) and lower odds of inability to obtain needed medical care (OR 0.17, 95% CI 0.06-0.47), although these associations were not significant among participants without a prior history of cancer.

**Conclusions:**

We describe widespread use of digital technologies in a community-based cohort, although disparities persist. In this cohort, smartphone ownership was significantly associated with ability to obtain needed medical care, suggesting that the use of smartphone technology may play a role in increasing health care access. Similarly, major illnesses such as cancer have the potential to amplify health engagement. Finally, special emphasis must be placed on reaching patient populations with limited digital access, so these patients are not further disadvantaged in the new age of telehealth.

## Introduction

### Background

The COVID-19 pandemic has highlighted the increasing dependence of the health system on telemedicine because providers have relied on telehealth to provide patient care while minimizing the risk of viral transmission [1-3]. Telehealth, or the use of technology—including the internet and mobile phones—to enable or improve health or health care [4,5], has expanded in scope and capabilities in recent decades. Patients can now access personal health information through patient portals [6], look up health information independently without professional medical guidance [7-11], and provide feedback to other consumers on their experiences with certain hospitals or providers through these media [12]. Studies have shown that the use of technology in the health sector can improve intervention efficacy, patient satisfaction, and, ultimately, clinical outcomes [13-17]. Technology has assumed a growing role within the health care landscape, with more than 60% of all US health care institutions using at least one form of telehealth [18], with some engaging in more telehealth visits than in-person visits [19].

Although telehealth has shown promise as a means to expand access to care [20,21], documented disparities persist among individuals who engage with health technology. The gap in access to technology based on social, physical, and societal factors is often referred to as the *digital divide* [22]. Previous work has characterized this divide extensively, with older, less-educated individuals having lower use of internet, mobile phone, and smartphone technologies [23-25] and preferring to receive health information through printed media compared with younger and more-educated individuals [26]. Furthermore, bridging the divide involves not only addressing gaps in physical access to technology, known as the *first digital divide*, but also the reliability of access and technological literacy, known as the *second digital divide* [27-30].

The second digital divide reflects the well-documented association between lower rates of phone and internet use with decreased health literacy [31-33]. This relationship seems to be multifaceted, with recent studies demonstrating an association not only between health literacy and the likelihood of technology adoption, but also between health literacy and the use of health information technology [34,35]. These findings allude to the evolving body of literature characterizing the relationship between digital technology and health outcomes. Preliminary studies have demonstrated that increased internet use can positively affect perceived health outcomes as well as the use of health care services. These differences in the outcomes, benefits, and impacts of technology use represent the *third digital divide* [30,36]. Work thus far describing the relationship between health literacy and health outcomes has led to mixed, inconclusive results [37].

A better understanding of the nature and ramifications of the digital divide is critical because patient populations who are already disadvantaged are at increased risk of being marginalized as the health system evolves to increasingly rely on telehealth to deliver care. Studies have already been published describing the widening inequity resulting from the health system’s increased reliance on telehealth as a result of the COVID-19 pandemic and the negative repercussions that most frequently and meaningfully affect vulnerable communities [38]. Furthermore, given the United States’ ongoing poor performance in health care access and affordability compared with peer developed nations [39], the US health care system is particularly vulnerable to widening disparities as a result of the stress of dealing with the COVID-19 pandemic.

### Objective

This study seeks to provide valuable data from a racially diverse cohort of North Carolina residents to further characterize the current state of digital technology use and to explore the relationship among technological access, health literacy, and health care access. Furthermore, this study builds on preliminary research assessing how major illnesses such as cancer relate to internet- and smartphone-use behaviors and their associations with markers of health literacy and health care access [40,41].

## Methods

### Study Design and Participants

This cross-sectional study used survey data derived from a community health assessment initiative, Project PLACE (Population Level Approaches to Cancer Elimination). The survey was administered to a targeted convenience sample of diverse populations across a predefined patient catchment area in proximity to a National Cancer Institute–designated comprehensive cancer center in North Carolina, and it aimed to oversample historically underserved communities [42]. Data were collected from April 2017 to December 2017. The study protocol was approved by the Duke University Institutional Review Board (#00062661).

Most of the participants were recruited from community organizations located in Durham, Wake, Vance, Alamance, and Johnston counties in central North Carolina. Community navigators worked in conjunction with 24 community partners to collect survey data using a multimodal approach. The community partners—comprising community organizations, faith organizations, community outreach programs, and a health clinic—distributed the surveys to their constituents at 47 different community events. Community partners received stipends (US $10 per survey, up to US $2000) for their collaboration. Survey participants were offered items valued at US $5 or less (eg, water bottle and tote bag) for their participation [43].

### Survey Design

The 91-item, self-administered survey was available in English, Spanish, and Chinese and could be completed on paper or on the web. The survey items included a combination of program-specific and pre-existing validated measures sourced from national surveys, including the Health Information National Trend Survey and the National Health Interview Study [44]. Data from select survey items that assessed sociodemographic factors, personal cancer history, patterns of mobile phone and internet use, and markers of health literacy and health care access were used in this study (Multimedia Appendix 1). The place of residence, metropolitan or nonmetropolitan, was captured with self-reported ZIP code that was coded using Rural-Urban Continuum Codes (RUCC) in which RUCC 1 to 3 were coded as metropolitan and RUCC 4 to 9 were coded as nonmetropolitan [45,46].

### Independent Variables

The primary independent variables included internet use and smartphone ownership. Internet use was captured with this question: “Do you ever go on-line to access the Internet or World Wide Web, or to send and receive e-mail?” Smartphone ownership was captured with this question: “Do you currently have a Smart phone such as an iPhone, Android, Blackberry or Windows phone?”

### Outcome Measures

The primary outcome measures were health care access and markers of health literacy. Health care access was captured with several items, including having a usual place of care when sick or in need of advice regarding health, the type of place attended for care, being unable to get care when needed, health insurance status, and participation in medical research. Markers of health literacy were captured with several additional items, including having looked for information on health topics, the source used for information on health topics, self-reported confidence in the ability to obtain health information, and self-reported understanding of numerical information (ie, numeracy). The variable “self-reported numeracy” was dichotomized by grouping survey responses 1-3 as “low self-reported numeracy” and survey responses 4-6 as “high self-reported numeracy.” The variable “confidence in ability to obtain health information if needed” was dichotomized with the survey responses “completely confident,” “very confident,” and “somewhat confident” grouped together compared with the grouping of “a little confident” and “not at all confident” (Multimedia Appendix 1).

### Statistical Analysis

Descriptive statistics were used to summarize the study sample. Continuous and categorical variables were summarized as median (IQR), where IQR is reported as first quartile value–third quartile value, and n (%), respectively, by smartphone ownership and internet use. Differences were tested using the chi-square test or Fisher exact test for categorical variables, as appropriate, and the two-tailed *t* test for continuous variables.

Logistic regression was used to estimate the association of smartphone ownership and internet use, respectively, with health care use and literacy variables after adjustment for sociodemographic factors both in the total study cohort and after stratifying by personal history of cancer. Covariates were selected based on univariate analysis (*P*<.10). Only respondents with complete data were included in each analysis, and effective sample sizes are indicated for each table and figure. No adjustments were made for multiple comparisons. Two-tailed tests were used for all analyses, and the threshold for significance was set at *P*<.05. All statistical analyses were conducted using SAS version 9.4 (SAS Institute).

## Results

### Participant Sociodemographic and Digital Technology Use Characteristics

A total of 2315 surveys were completed. Of these 2315 surveys, 2149 (92.83%) respondents answered all 3 questions pertaining to mobile phone ownership, smartphone ownership, and internet use. Demographic, personal health history, and smartphone and internet use characteristics are shown in Table 1.

**Table 1:** Baseline characteristics of study cohort (N=2149)^a^.

Characteristics		All respondents	Smartphone ownership							Internet use						
			No (n=349), n (%)	Yes (n=1800), n (%)	*P* value		Chi-square (*df*)		No (n=228), n (%)		Yes (n=1921), n (%)		*P* value		Chi-square (*df*)	
Age, median (IQR)		51 (38-65)	68 (58-76)	48 (36-61)	<.001		272.3 (1)		67 (55-76)		49 (37-63)		<.001		130.1 (1)	
**Gender, n (%)**						.32		N/A^b^						.99^c^		N/A
	Female	1319 (61.38)	216 (16.4)	1103 (83.62)					133 (10.1)		1186 (89.92)					
	Male	732 (34.06)	106 (14.5)	626 (85.52)					74 (10.1)		658 (89.89)					
	Other	8 (0.37)	0 (0)	8 (100)					0 (0)		8 (100)					
**Race and ethnicity, n (%)**						.001		19.1 (4)						.94		0.8 (4)
	Hispanic	300 (13.96)	52 (17.3)	248 (82.67)					29 (9.7)		271 (90.33)					
	Non-Hispanic Asian	202 (9.4)	17 (8.4)	185 (91.58)					19 (9.4)		183 (90.59)					
	Non-Hispanic Black	666 (30.99)	78 (11.7)	588 (88.29)					64 (9.6)		602 (90.39)					
	Non-Hispanic White	655 (30.48)	118 (18)	537 (81.98)					55 (8.4)		600 (91.6)					
	Other	82 (3.82)	10 (12.2)	72 (87.8)					8 (9.8)		74 (90.24)					
**Income adequacy, n (%)**						.02		5.8 (1)						.27		1.2 (1)
	Living comfortably or getting by on present income	1629 (75.8)	234 (14.4)	1395 (85.64)					156 (9.6)		1473 (90.42)					
	Finding it difficult or very difficult on present income	366 (17.03)	71 (19.4)	295 (80.6)					42 (11.5)		324 (88.52)					
**Education level, n (%)**						<.001		138.1 (2)						<.001		140.6 (2)
	High school or less	487 (22.66)	151 (31)	336 (68.99)					114 (23.4)		373 (76.59)					
	Post high school training or some college	535 (24.9)	84 (15.7)	451 (84.3)					48 (8.97)		487 (91.03)					
	College graduate or higher	1017 (47.32)	78 (7.7)	939 (92.33)					40 (3.9)		977 (96.07)					
**Occupational status, n (%)**						<.001		251.5 (4)						<.001		100.6 (4)
	Disabled	83 (3.86)	30 (36.1)	53 (63.86)					19 (22.9)		64 (77.11)					
	Employed	1192 (55.47)	77 (6.5)	1115 (93.54)					66 (5.5)		1126 (94.46)					
	Unemployed	89 (4.14)	18 (20.2)	71 (79.78)					5 (5.6)		84 (94.38)					
	Retired	456 (21.22)	163 (35.7)	293 (64.25)					93 (20.4)		363 (79.61)					
	Other	190 (8.84)	20 (10.5)	170 (89.47)					15 (7.9)		175 (92.11)					
**Insurance status, n (%)**						<.001		196.3 (3)						<.001		70.7 (3)
	Private	1048 (48.77)	56 (5.3)	992 (94.66)					51 (4.9)		997 (95.13)					
	Public	626 (29.13)	192 (30.7)	434 (69.33)					108 (17.3)		518 (82.75)					
	Insured, unknown type	95 (4.42)	18 (18.9)	77 (81.05)					14 (14.7)		81 (85.26)					
	None	266 (12.38)	44 (16.5)	222 (83.46)					27 (10.2)		239 (89.85)					
**Location type, n (%)**						<.001		39.1 (1)						<.001		24.4 (1)
	Metropolitan	1696 (78.92)	222 (13.1)	1474 (86.91)					143 (8.4)		1553 (91.57)					
	Nonmetropolitan	373 (17.36)	97 (26)	276 (73.99)					63 (16.9)		310 (83.11)					
**Personal history of cancer, n (%)**						<.001		35.8 (1)						.07		3.3 (1)
	No	1761 (81.95)	247 (14)	1514 (85.97)					175 (9.9)		1586 (90.06)					
	Yes	340 (15.82)	92 (27.1)	248 (72.94)					45 (13.2)		295 (86.76)					

^a^Test statistics and *df* are presented for the chi-square and *t* test *P* values only.

^b^N/A: not applicable.

^c^Fisher exact test *P* value.

**Figure 1:**
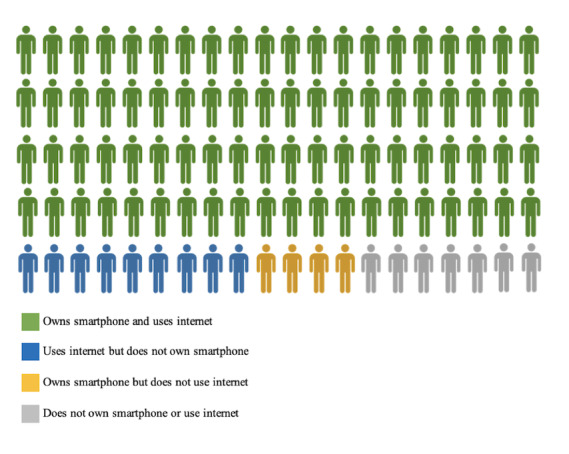
Prevalence of smartphone ownership and internet use among study cohort.

The median age was 51 years (IQR 38-65), and most of the participants were women (1319/2149, 61.38%). The racial and ethnic distribution was 13.96% (300/2149) Hispanic, 9.4% (202/2149) non-Hispanic Asian, 30.99% (666/2149) non-Hispanic Black, 30.48% (655/2149) non-Hispanic White, and 3.82% (82/2149) other, whereas 11.35% (244/2149) chose not to respond to this question. Overall, 75.8% (1629/2149) of the participants reported living comfortably or getting by on their present income, and 47.32% (1017/2149) reported an education level of college graduate or higher. Most of the participants lived in a metropolitan area (1696/2149, 78.92%), were employed (1192/2149, 55.47%), and did not have a history of cancer (1761/2149, 81.95%). In total, 89.39% (1921/2149) of the respondents reported using the internet, 96.32% (2070/2149) reported owning a mobile phone, and 83.76% (1800/2149) reported owning a smartphone (Figure 1).

### Sociodemographic Factors Associated With Digital Access

Specific characteristics distinguished smartphone owners and internet users from nonsmartphone owners and noninternet users (Table 1). Compared with smartphone owners and internet users, the respondents who reported not owning a smartphone or not using the internet were more likely to be older (both *P*<.001), less-educated (both *P*<.001), disabled (both *P*<.001), retired (both *P*<.001), publicly insured (both *P*<.001), and living in a nonmetropolitan area (both *P*<.001). The respondents who reported not owning a smartphone were also more likely to find it difficult or very difficult to get by on current income compared with smartphone owners (*P*=.02).

### Digital Technology Use and Markers of Health Literacy

Smartphone ownership and internet use demonstrated significant thematic associations with markers of health literacy (Table 2).

**Table 2:** Markers of health literacy and health access by smartphone ownership and internet use (N=2149).^a^

Survey response			All respondents, n (%)		Smartphone ownership											Internet use									
					No (n=349), n (%)		Yes (n=1800), n (%)		*P* value			Chi-square (*df*)			No (n=228), n (%)			Yes (n=1921), n (%)			*P* value			Chi-square (*df*)	
**Has looked for information on health or medical topics from any source**										.006			7.7 (1)									<.001			51.8 (1)
	No	249 (11.59)		55 (22.1)		194 (77.91)								59 (23.7)			190 (76.31)								
	Yes	1867 (86.88)		284 (15.2)		1583 (84.79)								164 (8.8)			1703 (91.22)								
**Is confident in ability to get health information if needed**										.001			10.9 (1)									.003			8.7 (1)
	Completely, very, or somewhat confident	1697 (78.97)		243 (14.3)		1454 (85.68)								139 (8.2)			1558 (91.81)								
	A little or not at all confident	142 (6.61)		35 (24.6)		107 (75.35)								22 (15.5)			120 (84.51)								
**Self-reported numeracy (1 is low, 6 is high**)										<.001			18.7 (1)									<.001			32.9 (1)
	1-3	710 (33.04)		146 (20.6)		564 (79.44)								110 (15.5)			600 (84.51)								
	4-6	1378 (64.12)		183 (13.3)		1195 (86.72)								103 (7.5)			1275 (92.53)								
**Has a usual place for health care or advice**										.37			1.9 (2)									.47			1.5 (2)
	Yes	1671 (77.76)		268 (16)		1403 (83.96)								173 (10.4)			1498 (89.65)								
	There is more than one place	164 (7.63)		26 (15.9)		138 (84.15)								14 (8.5)			150 (91.46)								
	There is no place	211 (9.82)		26 (12.3)		185 (87.68)								17 (8.1)			194 (91.94)								
**Usual place for health care**										.18			4.8 (3)									.35			3.2 (3)
	Hospital emergency room	63 (2.93)		14 (22.2)		49 (77.78)								8 (12.7)			55 (87.3)								
	Hospital outpatient department, clinic, or health center; doctor’s office or HMO^b^	1762 (81.99)		280 (15.9)		1482 (84.11)								178 (10.1)			1584 (89.9)								
	There is no one place	99 (4.61)		11 (11.1)		88 (88.89)								8 (8.1)			91 (91.92)								
	Some other place	40 (1.86)		9 (22.5)		31 (77.5)								7 (17.5)			33 (82.5)								
**Needed medical care but could not get in within the last 12 months**										<.001			17.8 (1)									.41			0.7 (1)
	No	1721 (80.08)		242 (14.1)		1479 (85.94)								165 (9.6)			1556 (90.41)								
	Yes	306 (14.24)		72 (23.5)		234 (76.47)								34 (11.1)			272 (88.89)								
**Asked to participate in a clinical trial or medical research**										.76			0.0 (1)									.002			9.2 (1)
	No	1599 (74.41)		255 (15.9)		1344 (84.05)								183 (11.4)			1416 (88.56)								
	Yes	494 (22.99)		76 (15.4)		418 (84.62)								33 (6.7)			461 (93.32)								

^a^Percentages may not add up to 100 owing to rounding or missing values.

^b^HMO: health maintenance organization.

On univariate analysis, both smartphone ownership and internet use were associated with higher self-reported numeracy (both *P*<.001), confidence in obtaining health information if needed (*P*=.001 and *P*=.003, respectively), and having looked for information on health topics from any source (*P*=.006 and *P*<.001, respectively).

**Figure 2:**
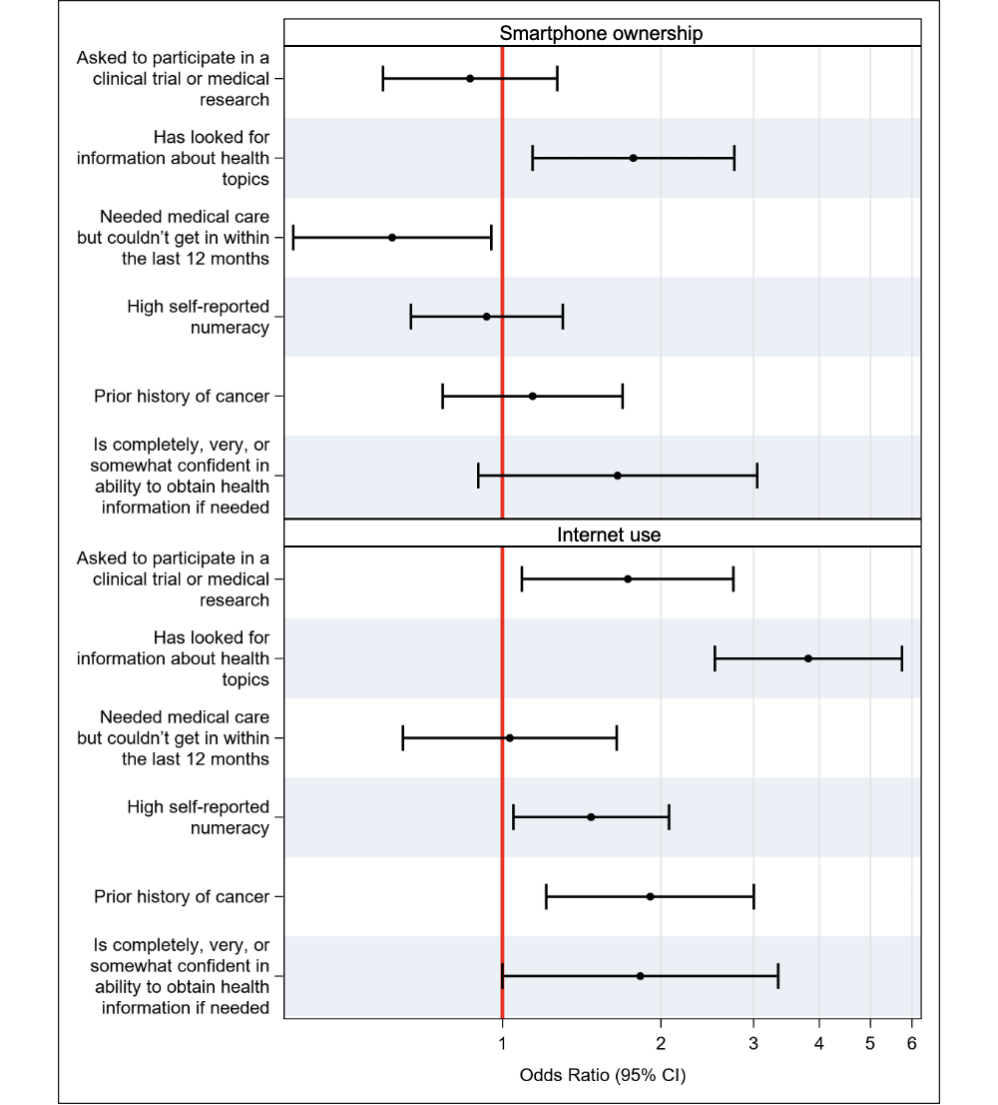
Association of markers of health literacy and health access with smartphone ownership (top) and internet use (bottom). Separate models were used for each outcome listed on the left, with smartphone ownership or internet use included as a covariate.

In the adjusted analysis, smartphone ownership was associated with higher odds of having looked for health information (odds ratio [OR] 1.77, 95% CI 1.14-2.76), when controlling for age, race and ethnicity, nativity, language spoken at home, income adequacy, occupational status, education level, insurance status, and rurality (Figure 2). Similarly, internet use was associated with higher odds of having looked for information on health topics (OR 3.81, 95% CI 2.53-5.75), confidence in obtaining health information if needed (OR 1.83, 95% CI 1.00-3.34), and self-reported numeracy (OR 1.47, 95% CI 1.05-2.07) when controlling for age, occupational status, education level, insurance status, and rurality (Figure 2). Smartphone ownership models were adjusted for the following covariates: age, race and ethnicity, nativity, spoken language, income adequacy, occupational status, education level, insurance status, and rurality. Covariates were selected based on univariate analysis (*P*<.10 on univariate analysis). Internet use models were adjusted for the following covariates: age, occupational status, education level, insurance status, and rurality. Covariates were selected based on univariate analysis (*P*<.10 on univariate analysis).

**Figure 3:**
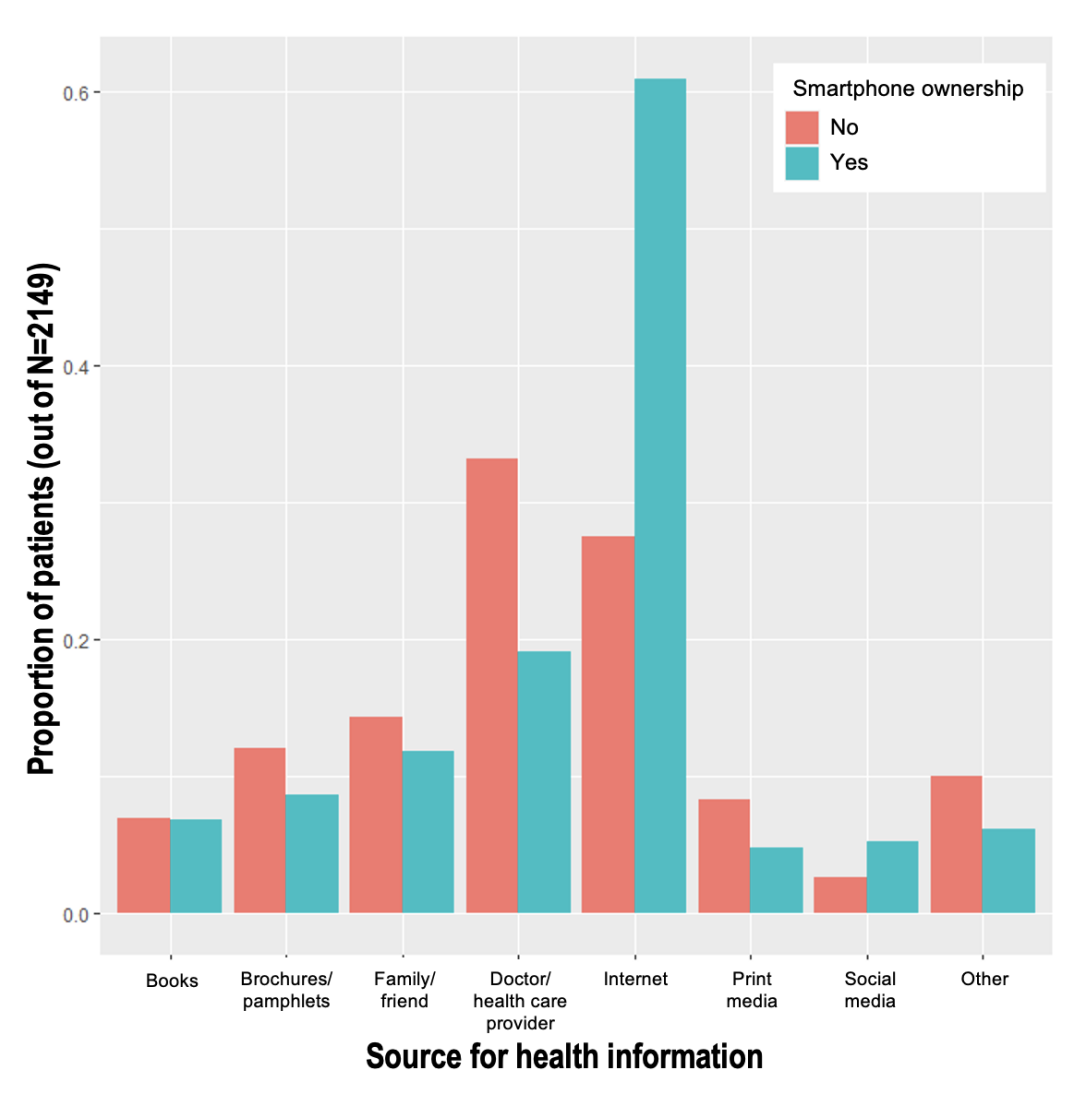
Source used most recently for health information by smartphone ownership.

**Figure 4:**
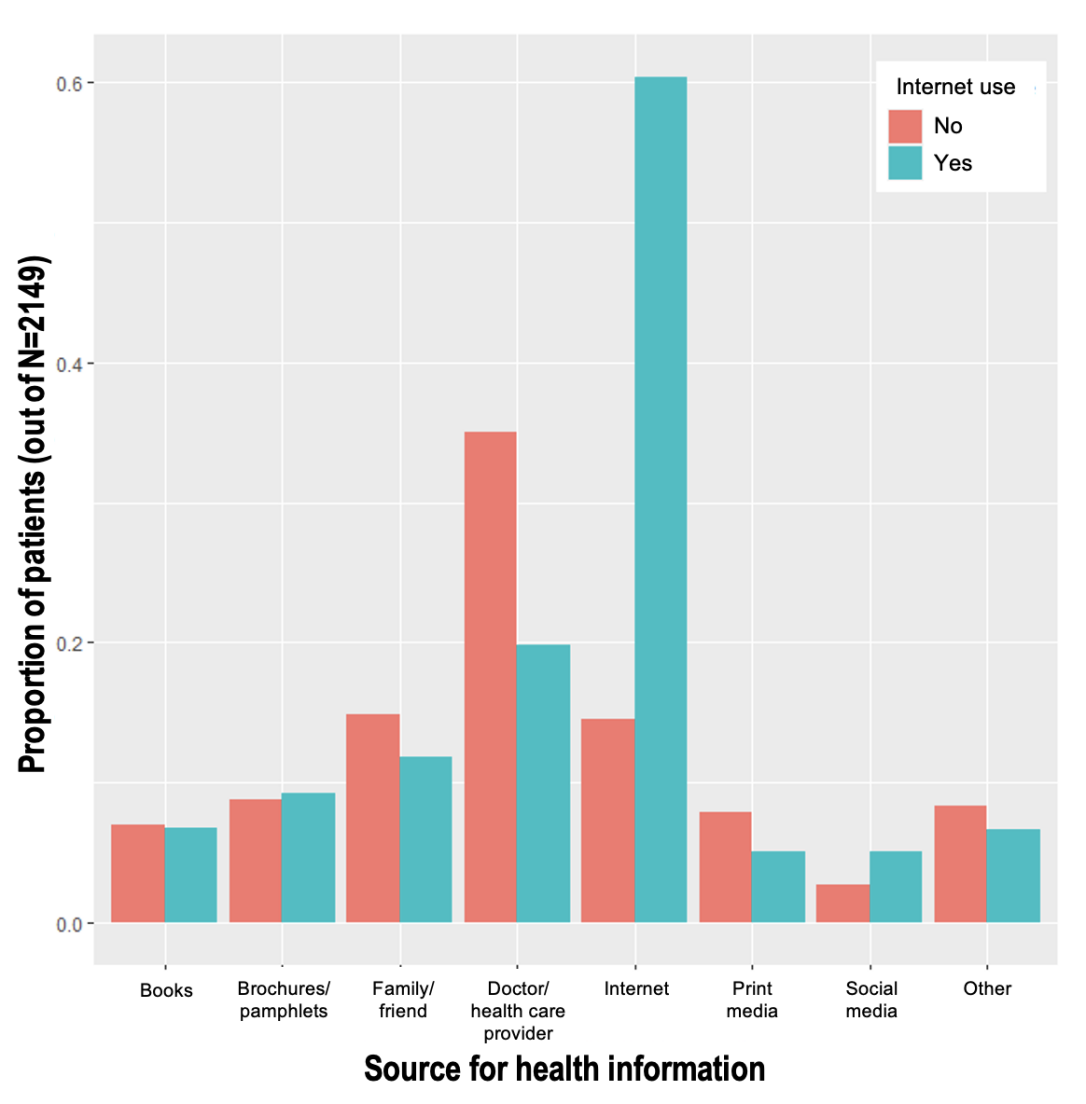
Source used most recently for health information by internet use.

These differences were reflected in the sources of information that the respondents reported using to obtain health information (Figures 3 and 4). Although most of the respondents did not use physicians as their most recent source of health information, the respondents without smartphones or internet access were more likely to have used a physician for this purpose (116/349, 33.2% of nonsmartphone owners vs 344/1800, 19.11% of smartphone owners; 80/228, 35.1% of noninternet users vs 380/1921, 19.78% of internet users; both *P*<.001). Similarly, nonsmartphone owners were less likely than smartphone owners to have used the internet (96/349, 27.5% vs 1098/1800, 61%, respectively; *P*<.001) or social media (9/349, 2.6% vs 93/1800, 5.17%, respectively; *P*=.04).

### Digital Technology Use and Health Access

Smartphone ownership and internet use were also associated with markers of health care access (Table 2). Neither smartphone ownership nor internet use was significantly associated with having a usual place for health care or with using an outpatient office or clinic more frequently than the emergency room. However, smartphone owners were less likely than nonsmartphone owners to have been unable to obtain needed medical care within the last 12 months (234/1800, 13% vs 72/349, 20.6%, respectively; *P*<.001).

In adjusted analyses (Figure 2), controlled for age, race and ethnicity, nativity, language spoken at home, income adequacy, occupational status, education level, insurance status, and rurality, this relationship remained significant (OR 0.62, 95% CI, 0.40-0.95). On univariate analysis, internet use was associated with users having been asked to participate in medical research (73.7%, *P*=.002), and this relationship also remained significant in the adjusted analyses (OR 1.73, 95% CI 1.09-2.75) when controlling for age, occupational status, education level, insurance status, and rurality (Figure 2).

### Digital Technology Use in Participants With a Personal History of Cancer

**Figure 5:**
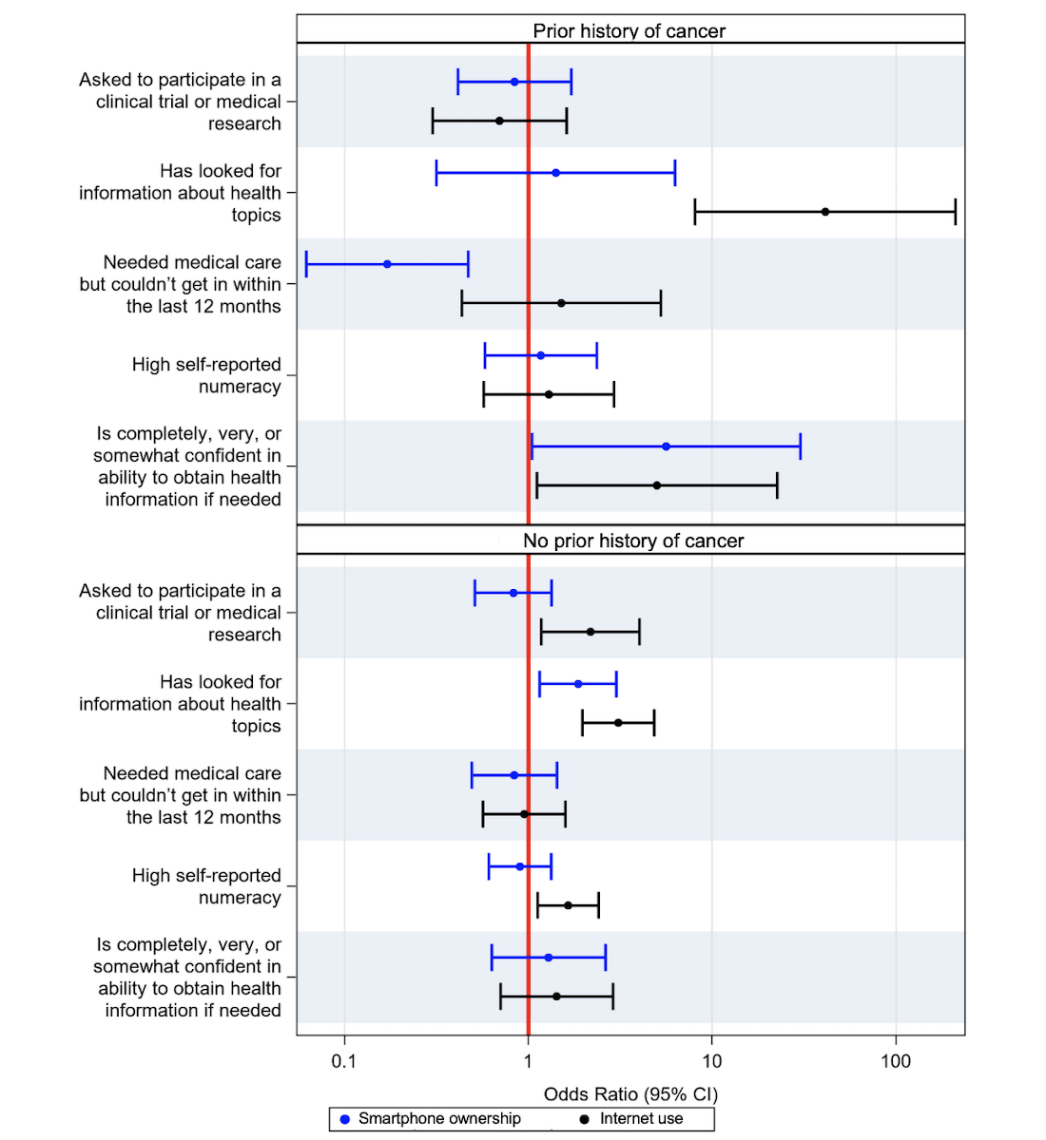
Association of smartphone ownership and internet use with markers of health literacy and health access in participants with (top) and without (bottom) a prior history of cancer. Separate models were used for each outcome listed on the left, with smartphone ownership or internet use included as a covariate.

The respondents who used the internet had higher odds of a personal history of cancer (OR 1.91, 95% CI 1.21-3.01; Figure 2). In adjusted analyses, older age (OR 1.06, 95% CI 1.05-1.07), disability (OR 4.12, 95% CI 2.30-7.37), and retired status (OR 1.64, 95% CI 1.08-2.49) were associated with an increased likelihood of a personal history of cancer. After stratifying the cohort by personal history of cancer, distinct relationships among smartphone ownership, internet use, and markers of health literacy and health access were observed. For participants with a personal history of cancer, smartphone owners had higher odds of being confident in their ability to obtain health information if needed (OR 5.63, 95% CI 1.05-30.23) and lower odds of needing medical care but being unable to get it (OR 0.17, 95% CI 0.06-0.47) compared with nonsmartphone owners, whereas these associations were not maintained among participants without a personal history of cancer (Figure 5). Similarly, among the participants with a history of cancer, internet users had higher odds of being confident in their ability to obtain health information if needed (OR 5.02, 95% CI 1.12-22.55), whereas this association was not maintained among participants without a prior history of cancer. All models were adjusted for the following covariates: age, occupational status, education level, insurance status, and rurality. Covariates were selected based on univariate analysis (*P*<.10 on univariate analysis).

## Discussion

### Principal Findings

Ensuring access to telehealth is an increasingly important priority for the medical community because the COVID-19 pandemic has shifted a large proportion of care into the digital realm, and the convenient and inexpensive nature of the medium suggests that its popularity will persist long after the pandemic eases [47]. As smartphone ownership and internet access are essential modes of digital connectivity in a world that increasingly relies upon such modalities to facilitate health care, smartphone and internet access could have significant implications for health outcomes. Prior studies describing patterns of connectivity are nearly a decade old, suggesting a gap in current evidence [48-50].

The rates of internet use, mobile phone ownership, and smartphone ownership in our cohort were high, with 89.39% (1921/2149) of the respondents reporting internet use, 96.32% (2070/2149) reporting mobile phone access, and 83.76% (1800/2149) reporting smartphone ownership. These rates are higher than prior estimates [51], highlighting the trend described in multiple studies of continued growth in digital usership [25,52,53]. Despite this increasing use, we found that a digital divide persists across socioeconomic dimensions [23,24]. Nonsmartphone and noninternet users were more likely to be older, disabled or retired, less well-educated, on public insurance, and residents of rural areas. Participants without smartphones were also more likely to report finding it difficult or very difficult to get by on their current income. These socioeconomic disparities in digital access have important implications. Both smartphone ownership and internet use were consistently associated with markers of health literacy. Importantly, our findings are the first to demonstrate an additional association between smartphone use and health care access. Finally, these data suggest that having a major medical condition, such as cancer, may serve to reverse previously described relationships between sociodemographic characteristics and technology use.

Smartphone and internet use were consistently and thematically associated with markers of health-specific and overall literacy [54]. Participants without smartphone or internet access had lower self-reported numeracy and lower confidence in their ability to obtain health information when needed. They also reported being less likely to look for information on health topics, even when controlling for potential sociodemographic confounders. These findings support an evolving body of literature that demonstrates an integral connection between digital access and health literacy. In a survey of 1077 patients at community health centers and outpatient clinics, Bailey et al [31] found that patients with adequate health literacy were more likely to own a mobile phone or smartphone and to have internet access. Similarly, a cross-sectional study of 131 low-income adults by Jensen et al [32] found that those with low health literacy skills were less likely to use the internet and related technologies. Low health literacy has been consistently associated with increased rates of hospitalization, greater use of the emergency department, increased medication nonadherence, decreased use of preventive services, and increased risk of mortality [55]. This body of data suggests that digital technology plays a key role in health outcomes.

Patient-provider communication may be an important mediator of the relationship between health literacy and health outcomes. In a survey of 823 patients presenting to an urban public hospital, Yin et al [56] found that patients with lower health literacy were more likely to rely on a doctor’s knowledge to make medical decisions and less likely to rely on their own knowledge or beliefs. Similarly, the respondents in our study who did not own smartphones or use the internet were significantly more likely to use a health care provider as their most recent source of health information. Importantly, the survey study by Yin et al [56] found that patients with lower health literacy were less likely to feel like partners in shared decision-making with their providers. These findings point to the need for both provider-level and systemic changes to provide increased support for those who rely on face-to-face health communication to inform their care. In addition, these findings highlight the need for further research into provider reliance on patients’ pre-existing medical knowledge or self-driven information seeking and how these behaviors may contribute to health disparities.

Our data are the first to demonstrate an association between smartphone ownership and disparities in access to care. Within our cohort, lack of smartphone ownership was significantly associated with being unable to obtain needed medical care, even when controlling for potential sociodemographic confounders, suggesting that characteristics associated with the use of digital technologies play a role in increasing health care access. Health literacy may be an important factor because prior studies have demonstrated an association between low health literacy and self-reported difficulty in accessing care [55,57]. However, health literacy is unlikely to be the only determinant because lack of internet use in our cohort was associated with lower markers of health literacy but was not significantly associated with being unable to obtain needed medical care. Taken together, these findings suggest that patients lacking smartphone access represent a unique subpopulation, distinct from those lacking internet access, and they may be the most vulnerable to the third digital divide [30] or the digital divide representing disparities in the impact of technology use on health outcomes. Indeed, even within our sample, nonsmartphone owners were distinct from noninternet users in key sociodemographic characteristics, including income adequacy. Future work should be directed toward identifying the unique barriers to care encountered by patients lacking smartphone access, including qualitative studies to assess patient experiences.

Finally, we found that a major health event such as a personal history of cancer may help overcome previously established sociodemographic patterns in digital technology use, with important implications for health outcomes. In our study, patients with a personal history of cancer were more likely to report internet use, despite being older than those without cancer, indicating that populations with historically low adoption of digital technologies, such as older patients [23], can be motivated to change their behavior when faced with a major illness. Importantly, our findings suggest that expanding digital access may also help to mitigate inequities in health care in this patient population. Among participants with a prior history of cancer, internet use and smartphone ownership were both significantly associated with confidence in being able to obtain needed health information. Smartphone use was also associated with lower odds of being unable to obtain needed medical care. In summary, the adoption of digital technologies in this patient population is associated with increased patient health activation, which in turn has been shown to have lasting benefits on health outcomes and health costs [58-60]. These findings demonstrate that engagement with the medical system in the form of a major illness can serve as a catalyst to overcome sociodemographic barriers to technology access, with the potential for improvement in long-term outcomes.

### Study Limitations

Our study included limitations that should be acknowledged. First, this was a cross-sectional study, and as such no conclusions can be drawn regarding the causal relationships among the variables studied. Second, the goals of this study were mainly exploratory; therefore, no adjustments were made for multiple comparisons in the statistical analyses. Third, the study findings are derived from self-reported data, which are subject to recall bias and may not accurately reflect participant health literacy and health care use. Fourth, there was a relatively small sample size of participants with a prior history of cancer who did not engage in smartphone or internet use, limiting our goal of precisely estimating the relationship between technology use and markers of health literacy and access in this cohort. The impact of cancer history on the relationship among digital technology, health literacy, and health access may be best explored through an alternative study design, such as a case-control study. In addition, these survey data were collected in the second half of 2017, and although they capture more recent trends in digital connectivity than those available in currently published literature, patterns of digital technology use have continued to evolve since these data were collected. Finally, this study cohort represented a convenience sample in which study recruitment was purposefully targeted to allow the study of a diverse cohort that allows for in-depth analysis of previously understudied patient populations. Thus, there may have been unmeasured biases in the study population, and the extent to which our findings may be generalizable to the national or statewide population is unknown. Future work, including large-scale population-based studies and qualitative investigations, will help to elucidate the connections among digital technology use, health literacy, and health access that were explored here.

### Conclusions

In conclusion, we found that access to digital technology has markedly increased across all social strata over the past decade in this diverse cohort of participants, leading to a more electronically connected society than ever before. This substantiates the claim that a broad digital infrastructure exists to support telemedicine as an increasingly important mode of patient engagement and health communication in the coming years. Nonetheless, a digital divide persists along sociodemographic and socioeconomic lines, with implications for both health literacy and access to care. Thus, dissemination of health technology must include measures to reach those with the most compromised health access. Importantly, our data support the idea that prolonged, meaningful contact with the health care system in the form of a major illness such as cancer has the potential to overcome sociodemographic trends in digital technology use and amplify the benefits these technologies confer. Future work should be directed toward the implementation of interventions to bridge the technology gap. In addition, more resources must be directed toward assisting vulnerable communities in engaging with applications of technology in the health sector because these applications hold promise in helping to mitigate health inequities and improve overall community health.

## Appendix

Multimedia Appendix 1 Participant survey items.

## Abbreviations

RUCC: Rural-Urban Continuum Code
OR: odds ratio
