# Supplementary material for: Association of Smartphone Ownership and Internet Use With Markers of Health Literacy and Access: Cross-sectional Survey Study of Perspectives From Project PLACE (Population Level Approaches to Cancer Elimination)
Source: J Med Internet Res. 2021 Jun 9;23(6):e24947. doi: 10.2196/24947 (PMC8262672; doi:10.2196/24947)
Supplement: Multimedia Appendix 1 [file jmir_v23i6e24947_app1.docx]

**Appendix 1.** Participant Survey Items

A1. Have you ever looked for information about health or medical topics from any source?

 Yes

 No

A2. The most recent time you looked for information about health or medical topics, where did you go first?

*Mark only one.*

 Books

 Brochures, pamphlets, etc.

 Cancer organization

 Family

 Friend/Co-worker

 Doctor or health care provider

 Internet

 Library

 Magazines

 Newspapers

 Telephone information number

 Complementary, alternative, or traditional medicine practitioner

 Social media site, such as *Facebook*

 Social media site for patient communities, such as *PatientsLikeMe*

 Other Specify: ______________________

Based on your most recent search for information about health, how much do you agree or disagree with the following statements?

A6. The information you found was hard for you to understand

 Strongly agree

 Somewhat agree

 Somewhat disagree

 Strongly disagree

A7. Overall, how confident are you that you could get advice or information about health or medical topics if you needed it?

 Completely confident

 Very confident

 Somewhat confident

 A little confident

 Not confident at all

A8. How often do you find numerical information easy to understand? By numerical information we mean information presented as numbers, tables or graphs.

 1  2  3  4  5  6

Never Very Often

B1. Do you own a cell phone?

 Yes

 No

B2. Do you currently have a Smart phone such as an iPhone, Android, Blackberry or Windows phone?

 Yes

 No

B4. Do you ever go on-line to access the Internet or World Wide Web, or to send and receive e-mail?

 Yes

 No

C2. Have you ever been diagnosed as having cancer?

 Yes

 No

D1. Have you ever been asked to participate in a clinical trial or medical research?

 Yes

 No

H1. Is there a place that you usually go to when you are sick or need advice about your health?

 Yes

 There is no place I usually go

 There is more than one place

H2. What kind of place do you go most often?

 Clinic or health center

 Doctor’s office or HMO

 A hospital emergency room

 A hospital outpatient department

 Some other place

 Doesn’t go to one place most often

H3. Was there a time in the past 12 months when you needed medical care, but could not get it?

 Yes

 No

N1. Are you male or female?

 Male

 Female

 Other Specify: _________________________

N3. What is your age?

 Years

N4. What is your current occupational status?

*Mark only one.*

 Employed – fulltime

 Employed – part time

 Unemployed

 Homemaker

 Student

 Retired

 Disabled

 Other-Specify ________________________

N6. What is the highest grade or level of schooling you completed?

 Less than 8 years

 Eight through 11 years

 12 years or completed high school

 Post high school training other than college (vocational or technical)

 Some college

 College graduate

 Postgraduate

N7. Were you born in the United States?

 Yes

 No

N9. What language do you speak at home?

 English

 Spanish

 Mandarin

 Bengali

 Cantonese

 Hindi

 Japanese

 Korean

 Taiwanese

 Telugu

 Vietnamese

 Other Specify: __________________

N10. Are you of Hispanic, Latino/a, or Spanish origin? Mark all that apply.

 Yes, Mexican, Mexican American, Chicano/a

 Yes, Puerto Rican

 Yes, Cuban

 Yes, Central American

 Yes, South American

 None of these

N11. What is your race? One or more categories may be selected. Mark all that apply.

 White

 Black or African American

 American Indian or Alaska Native

 Asian Indian

 Chinese

 Filipino

 Japanese

 Korean

 Vietnamese

 Other Asian

 Native Hawaiian

 Guamanian or Chamorro

 Samoan

 Other Pacific Islander

N12. Do you have any kind of health care coverage, including health insurance, prepaid plans such as HMOs, or government plans such as Medicare, or Indian Health Service?

 Yes

 No

N13. What is the primary source of your health care coverage? Is it…

 A plan purchased through an employer or union (includes plans purchased through another person's employer)

 A plan that you or another family member buys on your own

 Medicare

 Medicaid or other state program

 TRICARE (formerly CHAMPUS), VA, or Military

 Alaska Native, Indian Health Service, Tribal Health Services

 Some other source

N15. Which one of these phrases comes closest to your own feelings about your household’s income these days?

 Living comfortably on present income

 Getting by on present income

 Finding it difficult on present income

 Finding it very difficult on present income

N17. What is the ZIP Code where you currently live?


